# The Efficacy of the Global Intensive Feeding Therapy on Feeding and Swallowing Abilities in Children with Autism Spectrum Disorder: A Pilot Study

**DOI:** 10.3390/children10071241

**Published:** 2023-07-19

**Authors:** Antonella Cerchiari, Carolina Giordani, Silvia Franceschetti, Serena Mazzafoglia, Flavia Carosi, Francesca Pizza, Gessica Della Bella, Massimiliano Raponi, Marco Tofani

**Affiliations:** 1 Management and Diagnostic Innovations & Clinical Pathways Research Area, Neurorehabilitation and Adapted Physical Activity Day Hospital, Bambino Gesù Children’s Hospital, IRCCS, 00165 Rome, Italy; carolina.giordani@opbg.net (C.G.); silvia.franceschetti@opbg.net (S.F.); serena.mazzafoglia@opbg.net (S.M.); flavia.carosi@opbg.net (F.C.); francesca.pizza@opbg.net (F.P.); gessica.dellabella@opbg.net (G.D.B.); 2 Management and Diagnostic Innovations & Clinical Pathways Research Area, Medical Directorate, Bambino Gesù Children’s Hospital, IRCCS, 00165 Rome, Italy; massimiliano.raponi@opbg.net; 3 Management and Diagnostic Innovations & Clinical Pathways Research Area, Professional Development, Continuous Education and Research Service, Bambino Gesù Children’s Hospital, IRCCS, 00165 Rome, Italy; marco.tofani@uniroma1.it; 4 Department of Human Neurosciences, Sapienza University of Rome, 00183 Rome, Italy

**Keywords:** autism spectrum disorder, feeding, swallowing, behavior, children, chewing

## Abstract

The present investigation aims to explore the efficacy of Global Intensive Feeding Therapy (GIFT) on feeding and swallowing abilities in children with autism spectrum disorder (ASD). GIFT was developed as an intensive rehabilitation approach, divided into 30 sessions for 2 weeks, three times a day. GIFT focused on (a) encouraging desensitization; (b) widening the food repertoire (in terms of both variety and quantity); (c) reducing inappropriate mealtime behaviors; and (d) encouraging the development of appropriate chewing and swallowing abilities. GIFT was preliminarily implemented among 11 children with a diagnosis of ASD. To measure the efficacy of GIFT, the Karaduman Chewing Performance Scale (KCPS), the Brief Autism Mealtime Behavior Inventory (BAMBI), and food repertoire were investigated using Wilcoxon signed-rank test in three different times: baseline (T1), after treatment (T2), and one month after treatment (T3). Using Bonferroni correction, statistically significant differences were found between T1 and T2 for behavioral issues, as measured with BAMBI (*p* = 0.007), as well as for chewing abilities as measured with KCPS (*p* = 0.005) and for food acceptance (*p* = 0.005). These improvements were maintained after a month of follow-up, thanks to the collaboration of families and/or primary caregivers. In conclusion, GIFT seems to be an effective approach to improving behavioral issues, food acceptance, and chewing abilities in children with ASD.

## 1. Introduction

In children with autism spectrum disorder (ASD), the chances of presenting eating problems are five times higher than in the general pediatric population [1]. These feeding difficulties develop early and intensify over time, leading to a pattern of food selectivity, seen in more than 50% of the pediatric population with ASD.

Food selectivity is defined as a feeding disorder involving severe rigidity in food choices, which leads to a consumption of a limited variety of food, often lower than five, followed by a low acceptance of new meals [2]. Within the population with ASD, food selectivity is a rather significant issue: Several factors may be involved, including sensory modulation impairment [3,4], gastrointestinal disorders, motor and coordination impairment, restricted interests, repetitive and stereotyped behaviors, family upbringing, and habits; it has also been associated with inadequate dietary intake [5,6]. Thus, the lack of exposure to age-appropriate experiences fails to develop feeding abilities, as the motor patterns developed during feeding and sensory experiences are critical for the physiological development of the oral and facial structures and their functions. The specific characteristics of the sensory and motor profile in this population, such as hypersensitivity and the need for a rigorous routine, cause these children to forego the experience, preferring ‘simpler’ foods. This foresees difficulties in pursuing normal developmental stages and difficulties in later years, leading to the rejection of solid foods [7]. Moreover, children with ASD have different risk factors for poor nutrient intake associated with a selective diet, such as a lack of food variety [6].

Feeding difficulties can result in a negative impact on family life [8]. Sensory processing disorders can cause frustration during mealtime for both children and their families [9] and can affect the ability of children with ASD to behave in a socially acceptable way during mealtime in different settings [8,10]. Behavioral problems also play an important role in the eating habits of children with ASD: Their eating patterns tend to be driven by food aversion/rejection or preferences for certain types of food at the expense of others. Some factors involved include the texture, color, taste, shape, and temperature of the food; the shape and color of the packaging; the arrangement and presentation of dishes; and even the types of utensils used [11,12]. Sometimes, however, it is possible to identify the physiological factors that are the direct or indirect cause of specific eating and behavioral problems, including impaired sensory processing; delayed oral motor skills, such as chewing and swallowing; or gastric disorders [13].

The treatments of pediatric feeding disorders with the most empirical support in the research literature are based on applied behavior analysis (ABA) [14]. However, professionals often recommend, and caregivers often use, treatments that have limited empirical support [15], such as a sequential oral sensory approach to feeding and group feeding programs [16,17] or systematic desensitization [18]. To minimize the negative medical, nutritional, and behavioral impact of food selectivity, these children should be assessed by an interdisciplinary team to develop a child- and family-centered treatment plan [19].

The primary purpose of this study is to determine whether Global Intensive Feeding Therapy (GIFT), focused on the feeding abilities of children with ASD, can produce significant effects on food selectivity by enhancing these children’s acceptance of food, increasing their food repertoire, and facilitating the development of oral motor skills.

## 2. Materials and Methods

### 2.1. Design

The research group worked in the Feeding and Swallowing Disorder Service of the Bambino Gesù Children’s Hospital, IRCCS. We conducted a pilot study to assess the effectiveness of Global Intensive Feeding Therapy based on SLP intervention in terms of feeding and swallowing abilities in a sample of children with ASD and food selectivity.

### 2.2. Participants

We included children diagnosed with ASD having different levels of oral feeding abilities. Diagnosis of ASD was made by an expert MD specialized in child neuropsychiatry based on clinical evaluation and using Autism Diagnostic Observation Schedule (ADOS-2 protocol) [20]. Feeding abilities were evaluated by experienced speech and language pathologists throughout the clinical evaluation and using specific assessment tools, such as the Karaduman Chewing Performance Scale (KCPS) [21] (for further information, see the Assessment Tools, Outcome, and Procedures subsection). Children with comorbidity with other syndromes or medical disorders such as gastric disorders (gastroesophageal reflux and constipation) or dysphagia, and children undergoing behavioral therapy specific for feeding disorders were not included in this study. Any medical disorder (reflux, constipation) was handled before starting the rehabilitation treatment.

### 2.3. Treatment Program

#### 2.3.1. Setting

The room used during the rehabilitation treatment was designed to be as familiar and natural as possible for the children and their parents (with tables, a baby chair, a cupboard, a microwave, a fridge, a kitchen, etc.). As a part of the first session, parents were asked to bring their own assistive eating devices, toys (if preferred by the child at mealtimes), and food they knew their child would happily accept. The aim was to ease the gradual process of adjustment to the setting.

#### 2.3.2. Global Intensive Feeding Therapy (GIFT)

The GIFT rehabilitation program is built around the principles of neuroplasticity [22] and is individualized, so it has different features and is tailored to each child’s difficulties and needs. After clinical assessment, the children underwent an intensive rehabilitation treatment, which was developed into 30 sessions for 2 weeks, three times a day (breakfast, morning snack, and lunch). The intensive training is performed by a speech–language pathologist and the child’s parents. Parents must assist and learn specific techniques to use at home and help their child to generalize feeding and swallowing abilities. The intensive treatment period focuses on developing feeding and swallowing abilities while reducing dysfunctional behaviors. GIFT is focused on (a) encouraging desensitization; (b) widening the food repertoire (in terms of both variety and quantity); (c) reducing inappropriate mealtime behaviors; and (d) encouraging the development of appropriate chewing and swallowing abilities. To reach these objectives, the GIFT protocol can be summarized as follows:

***Systematic desensitization and gradual exposure.*** First, the work is focused on sensory issues, which will be the groundwork for the acceptance of food: The aim is to introduce the child to new food and help them gain familiarity with its smell, taste, shape, color, and texture. The child will build up confidence with food through a series of sensory stimulations according to a specific hierarchy, including visual tolerance, interaction, smell, and touch, starting from the most proximal areas of the body and gradually advancing to the perioral area and mouth, taste, and only at the end does the child proceed to eat the food itself [15,16,17]. Constant and intensive exposure to food stimuli is essential for it to be successful [23]. This can reduce the likelihood of reinforcing inappropriate mealtime behaviors [18]. The steps used for the acceptance of new food are described as follows: (1) to visually tolerate new food when placed directly in front of the child; (2) to touch the food, pick it up, and place it in another plate; (3) to pick up the food, smell it, and place it in another plate; (4) to pick up the food, kiss it, and place it in another plate; (5) to lick the food, making contact with the tongue; (6) to touch the food with teeth; (7) to bite the food with central incisors; and (8) to eat one small piece of the food. Each step is repeated until the child performs the required task on their own without engaging in any problem behavior. When the child completes a step, positive reinforcement is provided. To desensitize the posterior area of the oral cavity, the operator manually places the food on the posterior masticatory area, guiding the child in the chewing movement.

***Food Repertoire***. During two weeks of the program, new textures and flavors, and their differentiation (when the child only accepts a single meal) were explored, and the use of the baby bottle was reduced by asking the child to drink liquids from the glass while trying to increase the quantity of food accepted to ensure an appropriate nutritional intake. Moreover, it is essential to ensure the turnover of food options to avoid the reinforcement of food rigidity during mealtimes. The food for the children’s diet was selected based on the following process: (1) children who were bottle-fed at the initial assessment worked towards a reduction in the use of infant aid by replacing the bottle with the glass for drinking liquids; (2) transition from packed baby food to fresh mush; (3) introduction of fresh mush with divided flavors; (4) expansion of textures respecting systematic desensitization by gradual exposure; and (5) integrating soft solid foods through guided chewing training.

***Behavioral problems during mealtime.*** During the first assessment, the child’s behavior and the family’s management of the child during mealtimes are observed. Rules are set at the beginning of the treatment, respecting the child’s adjustment to the new setting. Positive and negative reinforcement and rewards are used to modulate disruptive behaviors for each feeding session; visual predictability strategies are also used. The child’s tolerance to frustration increased with gradual exposure; only when the child completes the feeding session does the demand increase by changing and adding the food offered.

***Chewing and Swallowing Abilities.*** Later, the treatment proceeds to chewing training, which allows for the consolidation or the development of the child’s oral motor abilities: It is an actual guided chewing practice. The underlying principle of this technique is that chewing is a learned ability [24]. Therefore, an improvement in chewing ability is possible with successful and repeated experiences [25].

Throughout this guided exercise, the child learns to control the mandibular movements and separate them from those of the tongue, inhibit the bite reflex, and use the muscular structures to encourage chewing. The tongue movements must separate from the jaw movements to develop tongue lateralization and posterization skills and encourage increased muscle tone, thus improving the ability to manage food in the oral cavity and saliva [26].

***Parent training.*** The caregiver is always involved throughout the treatment, being able to observe the techniques and strategies used by the operator by actively participating and choosing positive and negative reinforcements. The operator explains the desensitization techniques, the introduction of new foods, and how guided chewing training works. Initially, the parents observe and then gain direct experience with their child under the therapist’s supervision so that they can acquire the techniques and repeat them independently at home.

### 2.4. Assessment Tools, Outcome, and Procedures

For the present study, several assessment tools were used according to the objectives of investigating behavioral issues, as well as chewing performance and food repertoire:

**Brief Autism Mealtime Behavior Inventory (BAMBI).** The BAMBI [27] is an assessment tool developed as a response to the lack of sensitive instruments for measuring the eating behavior of children with ASD. The questionnaire was validated on children aged 3 to 11 years with and without ASD. The BAMBI is an observer-reported outcome measure specifically designed for parents and primary caregivers; it consists of 18 items scored using a five-level Likert scale. The BAMBI has good internal consistency (Cronbach’s α ranging from 0.79 to 0.88) and good test–retest reliability (ICC = 0.87) [27]. Besides the original version, the BAMBI was translated and validated into Brazilian Portuguese [28] and Turkish [29]. Lower scores indicate higher outcomes. For the present investigation, the Italian version of the BAMBI was used [30].

**Karaduman Chewing Performance Scale (KCPS).** The KCPS [31] is a valid, reliable, quick, and clinically easy-to-use functional instrument for determining the level of chewing function in children. Therefore, it was used to assess chewing function, allowing us to assign a score of 0 (normal chewing score), 1 (the child chews, but there are some difficulties in transitioning food to bolus), 2 (the child starts to chew, but he/she cannot hold the food in the molar area), 3 (the child bites but cannot chew), or 4 (the child cannot bite and chew). As this scale has not yet been translated and validated in Italian, the translation procedure has been performed independently from the English version, which was reported in the validation study by Serel Arslan and colleagues [31].

**Food Journal.** We conducted a parent interview focused on the child’s food acceptance before treatment, which was repeated at the end of the treatment and one month later (acceptance of food was defined as consuming food in good quantities in the absence of problem behavior). Food consistently seen in the child’s diet, and which did not trigger problem behavior was thus recorded and then classified according to its type (vegetables, bread, meat, etc.), type of preparation, quantity, and brand. In Italy, as well as at the international level [32,33], there is currently no validation of the construct of food selectivity among children with ASD that has reliable, reproducible methodologies and respects this population’s clinical features. Thus, in this study, children’s food selectivity was arbitrarily divided into the following groups: severe selectivity, those who accept 0–15 types of food; moderate selectivity, those who accept 15–40 types of food; and no selectivity, those accepting 40 or more types of food.

These assessment tools were used during the first assessment (T1), after two weeks of intensive treatment (T2), and at one-month follow-up (T3). To assess the feeding abilities after one month, the families were asked for a video of their child while having a meal, and the items of the rating scales were proposed via phone.

### 2.5. Data Analysis

The data from the anamnesis were analyzed using the frequency distribution to determine the number of times a particular value occurred in the dataset. Data of the BAMBI, KCPS, and food repertoire were analyzed at different times: before treatment (T1), after treatment (T2), and follow-up (T3). Data were analyzed using the Wilcoxon signed-rank test to investigate the differences among median values. The Wilcoxon signed-rank test is a frequently used nonparametric test for paired data consisting of pre- and post-treatment measurements based on independent units of analysis [34]. The significance-level alpha was set to <0.05. Our three hypotheses were tested using Bonferroni-adjusted alpha levels [35] of 0.016 per test (0.05/3): using the BAMBI, KCPS, and food repertoire, we hypothesized that GIFT can improve behavior, chewing performance, and acceptance of food.

## 3. Results

The study lasted 6 months and was conducted on a sample of 11 children (2 F and 9 M) with a mean age of 4.47 (ranging from 3 to 8 years old). All children participated in all training sessions during the two weeks of training. Sample characteristics, as well as chewing performance for each child, are reported in Table 1.

### 3.1. Behavioral Issues

Regarding behavioral issues, as measured with the BAMBI, a significant difference was found for the total score between T1 (mean 49.64 SD 12.38) and T2 (mean 35.09 SD 6.45), and between T1 and T3 (mean 33.45 SD 6.9), while no significant difference was found between T2 and T3 scores. Table 2 reports both the mean (SD) and median scores for each subscale of the BAMBI.

### 3.2. Chewing Abilities Issues

Concerning chewing abilities, as measured with the KCPS, a significant difference was found for the total score between T1 (mean 2.45 SD 1.63) and T2 (mean 1.55 SD 1.36) and between T1 and T3 (mean 1.45 SD 1.21), while no significant difference was found between T2 and T3 scores. Table 3 reports both the mean (SD) and median scores for the KCPS.

### 3.3. Food Repertoire

As for food repertoire, as measured by counting the number of accepted foods, a significant difference was found between T1 (mean 6.09 SD 3.48) and T2 (mean 20.36 SD 7.41) and between T1 and T3 (mean 29.57 SD 11.94), while no significant difference was found between T2 and T3 scores (*p* = 0.05). Table 4 reports both the mean (SD) and median scores for food repertoire.

## 4. Discussion

The present investigation provides preliminary evidence of GIFT in children with ASD in terms of chewing performance, behavioral issues, and food acceptance. Children were treated after an interdisciplinary assessment, providing evidence-based approaches for feeding and swallowing disorders [7,32,36,37].

### 4.1. Chewing Abilities

One of the main findings of our study is that children with ASD experience chewing difficulties. This issue is probably underestimated, and very few studies have confirmed that children with autism have a worse chewing function and worse mealtime functioning compared with typically developing children [24]. The KCPS was used to assess chewing skills, revealing that 82% of the sample had difficulties related to lateral tongue movements, breaking down food into small pieces efficiently, turning the food into bolus formation, and transferring it to the oropharynx. Furthermore, 45% had no chewing skills whatsoever (Level 4), regardless of the age of each subject, with difficulties in all the chewing stages. Among the children in the sample, some were still taking bottled milk after the physiological age and had absent chewing abilities; this is consistent with the literature reporting chewing difficulties in children who continue to use infant aids beyond the physiological age (3 years) [26]. Toddler ability persistence (sucking) also affects the amount of food and texture children accept. Furthermore, the data show that, if not appropriately treated, the delay in feeding skills persists even in older children (8 years), reflecting the recent scientific literature [38].

The outcomes observed by the end of the treatment (T2) indicate that working on prerequisites and developing oral motor and sensory skills leads to acquiring such abilities. An extremely significant improvement in chewing ability was observed after GIFT. In line with the concepts of motor learning [39], at the time of follow-up, we observed maintained motor and sensory oral skills, which was achieved through the constant work of parents, who were able to maximize the opportunities for practice in the everyday environment, extending the training to all five meals of the day. Their work allowed them to transfer learning possibilities from therapy to home contexts. In our study, only 18% of children were found to have age-appropriate chewing abilities (Level 0) when first assessed (T1); despite this, they had a limited repertoire and showed frequent disruptive mealtime behaviors. This highlights the fact that having good oral motor skills can still result in selective eating when not combined with appropriate mealtime behavior. We must therefore consider whether it is necessary to develop an integrated approach involving speech therapy alongside cognitive–behavioral work [32].

### 4.2. Food Repertoire

There is not one universally accepted mechanism through which feeding difficulties can be managed. A review of the previous literature focused on the aspect of food selectivity and increasing the food repertoire when managing feeding challenges [40]. All children increased the number of foods accepted after GIFT intervention (T2) (*p* = 0.003), showing an improvement even after one month (T3); however, this was not statistically significant (*p* = 0.05). This resulted from the work carried out during the two weeks of intensive training and later at home on feeding prerequisites and motor and sensory skills. By teaching the technique to caregivers, the child can undergo prolonged training to automatize motor skills (chewing) and sensory acceptance of new suggestions [22]. The involvement of parents during therapy helps them manage their child’s behavior, allowing the treatment to continue at home and therefore encouraging the generalization of learned abilities and the inclusion of new food. Qualitative considerations were taken into account. The textures accepted at the first assessment (T1) by the children in the sample were in line with their motor and oral sensory skills. This shows that food selectivity in children with ASD does not exclusively refer to the behavioral features associated with this syndrome but also to the absence of basic motor and oral sensory abilities. Several research studies have revealed that when there is food selectivity, people with ASD can fully benefit from adapted support aimed at both enhancing their intrinsic motivation and sensory functioning, as well as optimizing the physical and social environment, in order to make the experience of eating more enjoyable and more accessible [41].

The data showed a statistically significant difference between T1 and T2. Children accepted 6.09 types of food on average at the first assessment, whereas after the two weeks of treatment, they accepted 20.39 types of food on average. After one month, the improvement was not found to be statistically significant (*p* = 0.05), but from a qualitative point of view, an increased average number of food items was observed, increasing from an average of 20.36 to 29.57. These numbers support the importance of parental work and pursuing exercise in daily life. A further consideration concerns the children who took baby bottle milk beyond physiological age; using the bottle teat allowed them to bypass the oral cavity and trigger swallowing right from the oropharynx, thus excluding the oral sensory phase. This compromises the oral sensory experiences each child typically has and therefore impairs the normal development of feeding abilities [36].

### 4.3. Behavior during Mealtime

To analyze the frequency of dysfunctional mealtime behavior, the BAMBI was used [30]. However, this questionnaire mainly focuses on cognitive–behavioral aspects [18] and is less suitable for assessing in-depth feeding and swallowing abilities. For example, item n. 4 “The child spits out the food he or she has eaten” refers to the action “spitting” but does not allow us to explore its reasons. Despite the limitations when using the questionnaire, we found a significant difference regarding the reduction in dysfunctional mealtime behavior before and after the rehabilitation treatment (T1–T2), and a statistically significant difference was observed after follow-up, suggesting the consolidation of the progress made during the two-week training.

### 4.4. The Importance of Parent Involvement

The GIFT was followed by a monitoring period and a follow-up one month after the end of treatment. The best results during follow-up are achieved with constant functional training at home [25]. Parents, having actively participated in the intensive training, must continue with the work begun. They must repeat the techniques learned daily and apply them in different settings to consolidate and generalize the new abilities. In agreement with the guidelines of Assessment and Clinical Interventions for food selectivity in children with autism [32], parents are expected to play an active role throughout the child’s journey and must be the therapist’s first ally, and they must attend mealtimes to learn the feeding strategies and techniques to prevent or manage disruptive mealtime behaviors. This could help reduce parental stress [42], as disruptive mealtime behaviors, selective feeding, and child rigidity have been correlated with family stress and burden [43,44].

### 4.5. Differences between GIFT and a Cognitive–Behavioral Approach

Based on the sample’s inclusion criteria, no subjects had undergone specific SLP treatment for developing feeding and swallowing abilities. These findings agree with the current scientific literature, according to which feeding difficulties within this population are attributed to syndrome-specific features and, as such, can be re-educated with a cognitive–behavioral approach. In fact, rehabilitation treatments with greater empirical support involve the combination of behavioral and nutritional approaches [2,45]. However, these approaches aim to accept new food without considering the physiological development of feeding and swallowing abilities. This leaves out the underlying abilities required for the physiological development of feeding and swallowing abilities [7].

In the literature, feeding and swallowing disorders in this population are mostly included under the designation of “food selectivity,” which includes a wide range of behaviors (the presence of a restricted dietary pattern, decreased variety in food choice, food denial and piling, restricted caloric intake, preference for one type of food, problems during mealtime, and rituals and obsessions around food). These are mostly addressed from a behavioral, psychological viewpoint, without considering the fact that the origin of the listed behaviors can be of two different types: physiological (oral, peri-oral, and upper limb hypersensitivity or hyposensitivity; oral motor disorders; delayed feeding skills; and gastrointestinal disorders) and behavioral. Moreover, the origins of disruptive behaviors are not so separable categorically: many behaviors may result from physiological and/or behavioral issues triggered in a chain [36].

Chan and colleagues [46] revealed that most of the children with ASD who participated in their study had feeding disorders due to organic reasons, such as chewing disorders, choking, and oral motor and sensory disorders, which are all part of a speech–language pathologist’s competence. There are various approaches to support feeding in children with ASD, which include systematic desensitization, escape extinction, and reinforcement. Although these approaches are considered appropriate from a cognitive–behavioral point of view, they only address certain aspects of feeding disorders in children with ASD [46].

Only a few studies in the current literature address organic problems in feeding disorders in children with ASD [36]. However, we can consider feeding and swallowing disorders in these children as a consequence of their behavioral issues due to the syndrome only after we have ruled out organic problems [2]. Our study, centered on the clinical aspects of feeding and swallowing abilities, allows us to understand the multidimensional nature of such abilities better. The results indicate that a restricted repertoire in children with ASD is not only caused by cognitive–behavioral aspects. In fact, in children with ASD, it is common to observe delayed oral motor abilities (absence or gross chewing, permanent tongue suction, etc.), altered oral sensibility, and facial–oral dysfunctions (oral breathing, tongue thrust, drooling, articulation difficulties, and disphony). These dysfunctions can compromise feeding and swallowing development [13,47].

GIFT includes specific work on developing oral sensory and motor feeding skills to develop adequate feeding abilities. This is carried out by exposing the child to experiences through sensory games (e.g., touching, moving, smelling, and bringing food closer to the mouth), and then guided chewing training can induce the child to develop new motor patterns safely. The constant chewing training will help the child generalize this ability. Only with increased sensorimotor skills can the child increase his or her food repertoire, and only with sensory acceptance of different textures, smells, tastes, and temperatures can some dysfunctional behavior be contained and modified. Our SPL program (GIFT) involves a neurorehabilitation approach based on the principles of motor learning applied to eating and swallowing disorders. According to these concepts, the child must be subjected to practical tasks resembling the target skills to develop new learning (learning specificity). An adequate amount of training time is required to learn a skill: The intensiveness of rehabilitation enhances the number of repetitions useful for learning (distribution of practice) [39]. The caregiver’s use of rehabilitation techniques in the normal daily life activity of the meal increases the number of eating sessions and promotes the generalization of learning. The results show that caregivers’ presence in rehabilitation treatment is a key factor for successful treatment, in agreement with the current literature [46].

### 4.6. Study Limitations

It is important to consider some of the study’s limitations. Firstly, few articles in the current scientific literature report the results of SLP treatment in terms of feeding and swallowing abilities in children with ASD, which did not allow for an in-depth comparison among studies. Secondly, we did not have a group of comparison; therefore, as a pilot study, our results should be interpreted with caution. Thirdly, the absence of normative data on the number of foods accepted by children with normotypical development and children with ASD in the literature prevented us from using a standardized Italian assessment tool for evaluating food selectivity. Ultimately, children in the study were diagnosed through the ADOS-2 assessment [20], but the child’s level of functioning was not indicated. In the future, this information could be useful to observe whether there are specific criteria to be used in the treatment according to the level of functioning.

## 5. Conclusions

Global Intensive Feeding Therapy (GIFT) promotes chewing abilities, food acceptance, and behavioral issues, also involving families and primary caregivers. In conclusion, it is possible to state that GIFT seems to be an effective approach for children with ASD. Further research should confirm this preliminary efficacy with a larger sample and a different study design. Furthermore, other populations can also benefit from the GIFT approach; therefore, further exploration of this approach in other populations is highly recommended.

## Figures and Tables

**Table 1:** Sample characteristics.

Sample Characteristics	Total 11
Age mean (SD)	4.82 (1.47)
**Gender**	
Male	9
Female	2
**KCPS Level Distribution**	
Level 0: Normal chewing function	2 (18%)
Level 1: The child chews but there are some difficulties in transitioning food to bolus	1 (9%)
Level 2: The child starts to chew but he/she cannot hold the food in the molar area	3 (28%)
Level 3: The child bites but cannot chew	0 (0%)
Level 4: The child cannot bite and chew	5 (45%)
**KCPS mean (SD) score**	2.45 (1.63)
**Food repertoire mean (SD) score**	6.09 (3.48)
**BAMBI mean (SD) score**	49.64 (12.38)

KCPS: Karaduman Chewing Performance Scale; BAMBI: Brief Autism Mealtime Behavior Inventory.

**Table 2:** Differences in BAMBI scores in different phases of the intervention.

**BAMBI**	**T1 Mean (SD)**	**T2 Mean (SD)**	**Median T1**	**Median T2**	**Sig**
Limited Variety	26.82 (8.5)	17.18 (4.6)	27.00	16.00	0.007 *
Food Refusal	11.55 (4.4)	9.5 (3.38)	12.00	9.00	0.11
Features of Autism	11.27 (3.46)	8.45 (2.06)	11.00	9.00	0.011 *
Total	49.64 (12.38)	35.09 (6.45)	50.00	33.00	0.005 *
**BAMBI**	**T2 Mean (SD)**	**T3 Mean (SD)**	**Median T2**	**Median T3**	**Sig**
Limited Variety	17.18 (4.6)	16.73 (3.8)	16.00	16.00	0.67
Food Refusal	9.5 (3.38)	8.82 (2.9)	9.00	9.00	0.30
Features of Autism	8.45 (2.06)	7.91 (2.46)	9.00	8.00	0.234
Total	35.09 (6.45)	33.45 (6.9)	33.00	31.00	0.235
**BAMBI**	**T1 Mean (SD)**	**T3 Mean (SD)**	**Median T1**	**Median T3**	**Sig**
Limited Variety	26.82 (8.5)	16.73 (3.8)	27.00	16.00	0.008 *
Food Refusal	11.55 (4.4)	8.82 (2.9)	12.00	9.00	0.038
Features of Autism	11.27 (3.46)	7.91 (2.46)	11.00	8.00	0.012 *
Total	49.64 (12.38)	33.45 (6.9)	50.00	31.00	0.007 *

* Bonferroni-adjusted alpha level *p* < 0.016; BAMBI: Brief Autism Mealtime Behavior Inventory.

**Table 3:** Differences in KCPS scores in different phases of the intervention.

Karaduman Chewing Performance Scale (KCPS)						
	T1	T2		T1	T2	Sig
**mean (SD)**	2.45 (1.63)	1.55 (1.36)	**Median**	2.00	1.00	0.008 *
	**T2**	**T3**		**T2**	**T3**	**Sig**
**mean (SD)**	1.55 (1.36)	1.45 (1.21)	**Median**	1.00	1.00	0.317
	**T1**	**T3**		**T1**	**T3**	**Sig**
**mean (SD)**	2.45 (1.63)	1.45 (1.21)	**Median**	2.00	1.00	0.005 *

* Bonferroni-adjusted alpha level *p* < 0.016.

**Table 4:** Differences in acceptance of food in different phases of the intervention.

Food Repertoire						
	T1	T2		T1	T2	Sig
**mean (SD)**	6.09 (3.48)	20.36 (7.41)	**Median**	6	21	0.003 *
	**T2**	**T3**		**T2**	**T3**	**Sig**
**mean (SD)**	20.36 (7.41)	29.57 (11.94)	**Median**	21	27	0.05
	**T1**	**T3**		**T1**	**T3**	**Sig**
**mean (SD)**	2.45 (1.63)	29.57 (11.94)	**Median**	6	27	0.005 *

* Bonferroni-adjusted alpha level *p* < 0.016.

## Data Availability

Data are available from the corresponding author upon reasonable request.
